# Are Markers of Inflammation More Strongly Associated with Risk for Fatal Than for Nonfatal Vascular Events?

**DOI:** 10.1371/journal.pmed.1000099

**Published:** 2009-06-23

**Authors:** Naveed Sattar, Heather M. Murray, Paul Welsh, Gerard J. Blauw, Brendan M. Buckley, Stuart Cobbe, Anton J. M. de Craen, Gordon D. Lowe, J. Wouter Jukema, Peter W. Macfarlane, Michael B. Murphy, David J. Stott, Rudi G. J. Westendorp, James Shepherd, Ian Ford, Chris J. Packard, for the Prospective Study of Pravastatin in the Elderly at Risk (PROSPER) Study Group

**Affiliations:** 1 Division of Cardiovascular and Medical Sciences, Faculty of Medicine, University of Glasgow, Glasgow, Scotland; 2 Robertson Centre for Biostatistics, University of Glasgow, Glasgow, Scotland; 3 Section of Gerontology and Geriatrics, Leiden University Medical Centre, Leiden, The Netherlands; 4 Department of Pharmacology and Therapeutics, Cork University Hospital, Wilton, Cork, Ireland; 5 Departments of Cardiology, Leiden University Medical Centre, Leiden, The Netherlands; The George Institute, Australia

## Abstract

**Background:**

Circulating inflammatory markers may more strongly relate to risk of fatal versus nonfatal cardiovascular disease (CVD) events, but robust prospective evidence is lacking. We tested whether interleukin (IL)-6, C-reactive protein (CRP), and fibrinogen more strongly associate with fatal compared to nonfatal myocardial infarction (MI) and stroke.

**Methods and Findings:**

In the Prospective Study of Pravastatin in the Elderly at Risk (PROSPER), baseline inflammatory markers in up to 5,680 men and women aged 70–82 y were related to risk for endpoints; nonfatal CVD (i.e., nonfatal MI and nonfatal stroke [*n* = 672]), fatal CVD (*n* = 190), death from other CV causes (*n* = 38), and non-CVD mortality (*n* = 300), over 3.2-y follow-up. Elevations in baseline IL-6 levels were significantly (*p* = 0.0009; competing risks model analysis) more strongly associated with fatal CVD (hazard ratio [HR] for 1 log unit increase in IL-6 1.75, 95% confidence interval [CI] 1.44–2.12) than with risk of nonfatal CVD (1.17, 95% CI 1.04–1.31), in analyses adjusted for treatment allocation. The findings were consistent in a fully adjusted model. These broad trends were similar for CRP and, to a lesser extent, for fibrinogen. The results were also similar in placebo and statin recipients (i.e., no interaction). The C-statistic for fatal CVD using traditional risk factors was significantly (+0.017; *p*<0.0001) improved by inclusion of IL-6 but not so for nonfatal CVD events (*p* = 0.20).

**Conclusions:**

In PROSPER, inflammatory markers, in particular IL-6 and CRP, are more strongly associated with risk of fatal vascular events than nonfatal vascular events. These novel observations may have important implications for better understanding aetiology of CVD mortality, and have potential clinical relevance.

*Please see later in the article for Editors' Summary*

## Abstract 2

In a secondary analysis of a randomized trial comparing pravastatin versus placebo for the prevention of coronary and cerebral events in an elderly at-risk population, Naveed Sattar and colleagues find that inflammatory markers may be more strongly associated with risk of fatal vascular events than nonfatal vascular events.

## Editors' Summary

**Background:**

Cardiovascular disease (CVD)—disease that affects the heart and/or the blood vessels—is a common cause of death in developed countries. In the USA, for example, the leading cause of death is coronary heart disease (CHD), a CVD in which narrowing of the heart's blood vessels by “atherosclerotic plaques” (fatty deposits that build up with age) slows the blood supply to the heart and may eventually cause a heart attack (myocardial infarction). Other types of CVD include stroke (in which atherosclerotic plaques interrupt the brain's blood supply) and heart failure (a condition in which the heart cannot pump enough blood to the rest of the body). Smoking, high blood pressure, high blood levels of cholesterol (a type of fat), having diabetes, and being overweight all increase a person's risk of developing CVD. Tools such as the “Framingham risk calculator” take these and other risk factors into account to assess an individual's overall risk of CVD, which can be reduced by taking drugs to reduce blood pressure or cholesterol levels (for example, pravastatin) and by making lifestyle changes.

**Why Was This Study Done?:**

Inflammation (an immune response to injury) in the walls of blood vessels is thought to play a role in the development of atherosclerotic plaques. Consistent with this idea, several epidemiological studies (investigations of the causes and distribution of disease in populations) have shown that people with high circulating levels of markers of inflammation such as interleukin-6 (IL-6), C-reactive protein (CRP), and fibrinogen are more likely to have a stroke or a heart attack (a CVD event) than people with low levels of these markers. Although these studies have generally lumped together fatal and nonfatal CVD events, some evidence suggests that circulating inflammatory markers may be more strongly associated with fatal than with nonfatal CVD events. If this is the case, the mechanisms that lead to fatal and nonfatal CVD events may be subtly different and knowing about these differences could improve both the prevention and treatment of CVD. In this study, the researchers investigate this possibility using data collected in the Prospective Study of Pravastatin in the Elderly at Risk (PROSPER; a trial that examined pravastatin's effect on CVD development among 70–82 year olds with pre-existing CVD or an increased risk of CVD because of smoking, high blood pressure, or diabetes).

**What Did the Researchers Do and Find?:**

The researchers used several statistical models to examine the association between baseline levels of IL-6, CRP, and fibrinogen in the trial participants and nonfatal CVD events (nonfatal heart attacks and nonfatal strokes), fatal CVD events, death from other types of CVD, and deaths from other causes during 3.2 years of follow-up. Increased levels of all three inflammatory markers were more strongly associated with fatal CVD than with nonfatal CVD after adjustment for treatment allocation and for other established CVD risk factors but this pattern was strongest for IL-6. Thus, a unit increase in the log of IL-6 levels increased the risk of fatal CVD by half but increased the risk of nonfatal CVD by significantly less. The researchers also investigated whether including these inflammatory markers in tools designed to predict an individual's CVD risk could improve the tool's ability to distinguish between individuals with a high and low risk. The addition of IL-6 to established risk factors, they report, increased this discriminatory ability for fatal CVD but not for nonfatal CVD.

**What Do These Findings Mean?:**

These findings indicate that, at least for the elderly at-risk patients who were included in PROSPER, inflammatory markers are more strongly associated with the risk of a fatal heart attack or stroke than with nonfatal CVD events. These findings need to be confirmed in younger populations and larger studies also need to be done to discover whether the same association holds when fatal heart attacks and fatal strokes are considered separately. Nevertheless, the present findings suggest that inflammation may specifically help to promote the development of serious, potentially fatal CVD and should stimulate improved research into the use of inflammation markers to predict risk of deaths from CVD.

**Additional Information:**

Please access these Web sites via the online version of this summary at http://dx.doi.org/10.1371/journal.pmed.1000099.

- The MedlinePlus Encyclopedia has pages on coronary heart disease, stroke, and atherosclerosis (in English and Spanish)
- MedlinePlus provides links to many other sources of information on heart diseases, vascular diseases, and stroke (in English and Spanish)
- Information for patients and caregivers is provided by the American Heart Association on all aspects of cardiovascular disease, including information on inflammation and heart disease
- Information is available from the British Heart Foundation on heart disease and keeping the heart healthy
- More information about PROSPER is available on the Web site of the Vascular Biochemistry Department of the University of Glasgow

## Introduction

Low-grade chronic inflammation is now widely thought to play an important role in the process of atherogenesis [1], and levels of inflammatory markers such as C-reactive protein (CRP) [2], interleukin (IL)-6 [3], and fibrinogen [4] are moderately associated with risk of coronary heart disease (CHD) events. Recently we reported that whilst CRP was associated with subsequent combined nonfatal and fatal vascular events in the elderly at risk, the C-statistic was moderately (albeit significantly) increased with addition of CRP to traditional risk factors [5]. Other studies have reported similar findings in that all inflammatory markers appear to have moderate associations with risk of CHD events [6],[7], and provide minimal clinical utility to the C-statistic in risk scores [8]–[10].

Often in epidemiological studies, fatal and nonfatal cardiovascular disease (CVD) events are combined for the purposes of increasing power in a single endpoint. Obviously these CVD events share common aetiology, but the question may be asked as to whether the nature of the event differs only in severity or whether there are subtle differences in underlying mechanisms that lead to fatal events versus nonfatal outcomes. Insensitivities to such clinically important questions in many reports through study design, limited endpoint acquisition, and analysis may be one reason that acute phase response-associated inflammatory risk markers predict CVD events moderately in many prospective studies.

Comprehensive investigation of these questions is required to extend trends noted, but rarely commented upon, in other studies. For instance, in the Scottish Heart Health Study, plasma fibrinogen showed at least as strong an association with total mortality as with risk of CHD [11], a finding subsequently confirmed in the Fibrinogen Studies Collaboration [4]. Similar findings have been reported for blood viscosity (of which fibrinogen is a determinant) [12], IL-6, and CRP [13]–[20]. These potential differences and their implications are not widely appreciated by the vascular community. Additionally, simultaneous and comprehensive investigation of associations of these separate endpoints, including in particular separation of fatal and nonfatal CVD, with a range of inflammatory markers is required but is lacking. Potential differences may have implications both for our understanding of the role of inflammation in CVD aetiology, and for the development of future risk scores based on risk for fatal CVD events.

In PROSPER [21], a clinical trial of pravastatin in the elderly at risk, with more than 850 incident CVD events including 190 fatal CVD events, we had the potential to explore for differential associations of IL-6, CRP, and fibrinogen with subsequent prespecified vascular events (fatal versus nonfatal), as well as examine whether such associations were independent of traditional risk factors.

## Methods

The protocol of PROSPER has been published elsewhere [21],[22], and the methodology and outcome of the main trial has also been published [22].

### Participants

Between December 15, 1997, and May 7, 1999, we screened and enrolled individuals from Scotland, Ireland, and The Netherlands. Briefly, men and women aged 70–82 y were recruited if they had either preexisting vascular disease (coronary, cerebral, or peripheral) or increased risk of such disease because of smoking, hypertension, or diabetes. Their plasma total cholesterol was required to be 4.0–9.0 mmol/l and their triglyceride concentrations less than 6.0 mmol/l. After screening, eligible individuals entered a 4-wk single-blind placebo lead-in period, those who used <75% or >120% of the placebo medication were excluded. The institutional ethics review boards of all centres approved the protocol, and all participants gave written informed consent. The protocol was consistent with the Declaration of Helsinki.

### Protocol

Individuals were randomized to receive either pravastatin 40 mg daily or matching placebo [21].We reviewed participants every 3 mo, with a mean of 3.2-y follow-up. Lipoprotein profiles were measured at the Centre for Disease Control-certified central lipoprotein laboratory in Glasgow. All data were processed and analysed at the study data centre in The Robertson Centre for Biostatistics, Glasgow.

The outcomes of the present analyses were predefined as: (1) nonfatal CVD: combined endpoints of nonfatal myocardial infarction (MI) and nonfatal stroke (comprises infarcts and haemorrhages); (2) fatal CVD: definite or suspect death from CHD or stroke (comprises infarcts and haemorrhages); (3) fatal other CVD: definite or suspect death from “other” CVD causes (aneurysm, pulmonary embolism, heart failure); (4) non-CVD mortality: death from non-CVD causes.

For CVD events only the first event was included (i.e., deaths preceded by a nonfatal event were excluded and participants were censored at date of nonfatal event). All endpoints were validated by a blinded events committee.

### Laboratory Aspects

Baseline lipids, blood pressure, and body mass index (BMI) were measured as previously reported [21],[22]. IL-6 was assayed on a saved biobank of baseline samples in 2007 using a high-sensitivity ELISA (R& D Systems) with inter- and intra-assay CVs of <6% and a sensitivity of <0.16 pg/ml. CRP was measured as detailed previously [5] on an assay with a sensitivity of <0.17 mg/dl and with inter- and intra-assay CVs of <5%. Fibrinogen was determined by the Clauss method on an MDA 180 coagulometer (Trinity Biotech) and using the 9th British Standard to calibrate (NIBSC), with intra- and inter-assay CVs of <4%. All samples were processed by technicians blinded to the identity of samples, and results entered into a master database, which the PROSPER statistics division in Scotland (Robertson Centre, University of Glasgow) is custodian of.

### Statistical Analysis

The distribution of IL-6 (pg/ml) and CRP (mg/dl) was positively skewed and a logarithmic transformation was used. Baseline characteristics were compared between participants with and without the primary endpoint (as defined in original study) using the two-sample *t*-test for continuous variables and the chi-squared test for categorical variables. The influence of IL-6, CRP, and fibrinogen on the endpoints of interest—extended herein to include nonfatal CVD events, fatal CVD events, fatal other CV events, or non-CVD death—were investigated using Kaplan-Meier curves (for IL-6) and Cox proportional hazards models, adjusting for randomized treatment and baseline covariates (continuous variables: age, low-density lipoprotein [LDL] cholesterol, high-density lipoprotein [HDL] cholesterol, blood pressure, BMI, log triglyceride; categorical variables: gender, country, smoking status, history of diabetes, use of antihypertensive therapy, history of CHD, history of peripheral arterial disease [PAD], and history of stroke or TIA). Log IL-6, log CRP, and fibrinogen were analysed by 1-unit increments within the population, and hence represent a continuous measure, providing maximum power for detecting an association. Results are reported as number (percentage [%]) of events and hazard ratio (HR) of a given endpoint (95% confidence interval [CI]) for each unit increment. The validity of the proportional hazards assumption was assessed by plots of Cox-Snell residuals and Martingale residuals with no evidence of significant departures observed. In addition, IL-6 associations with endpoints were also analysed by comparing extreme thirds of the population based on IL-6 expression in the whole cohort. A similar method was previously reported for CRP [3]. A competing risks model was constructed to test the difference in the HRs for nonfatal versus fatal CVD events. The model combined nonfatal CVD and fatal CVD events, and treated all other deaths as censored [23],[24]. C-statistics (analogous to the area under the ROC curve) were calculated for Cox proportional hazards survival models and with and without adjustment for log IL-6 or CRP as continuous measures and are reported for selected models, along with probability values testing whether the inclusion of log IL-6 or CRP lead to predictions that are more concordant with observed events. Summary statistics are reported as mean (standard deviation [SD]) for continuous variables and number (%) for categorical variables. A *p*-value of <0.05 was considered significant.

## Results

Plasma IL-6 levels were obtained in 5,653 biobank samples (97.4%) of the original 5,804 patients. Of these, 667 (11.8%) had a nonfatal CVD event, 189 (3.3%) had a fatal CVD event, 37 (0.7%) died from other CVD causes (including 18 aneurysms, seven pulmonary embolisms, and three heart failures), and a further 299 (5.3%) had non-CVD deaths. Corresponding samples measured for CRP were 5,680 samples, 672 nonfatal CVD events, 190 fatal CVD events, 38 fatal other CVD events, and 300 non-CVD deaths. Fibrinogen was measured in 5,631 individuals. All other risk factor measurements were available in all individuals. 59 patients who had an incident nonfatal CVD went on to die from other events (vascular or otherwise). For the purpose of the present analysis, only the first event was included.

Baseline characteristics of participants who did and did not have a primary endpoint (defined in the original trial [22] and previous CRP analysis [5] as “CHD or stroke death or nonfatal MI or stroke”) are shown in Table 1. This table shows results broadly expected in an elderly at risk cohort, with significant differences between cases and noncases (i.e., those without an event) in many (but not all) conventional risk factors. Participants who had a primary vascular event during study follow-up were slightly older (6 mo on average), more likely to be male, and to have a history of diabetes, CHD, peripheral arterial disease, stroke or TIA, but less likely to have been taking pravastatin. Baseline HDL cholesterol, but not triglyceride or LDL cholesterol, were lower among subsequent cases. As expected, levels of IL-6 and CRP were also higher among the case than control population. Fibrinogen levels were not significantly different in the primary endpoint group. IL-6 concentrations were generally higher than levels seen in other middle-aged cohorts [3], but levels are in keeping with older age groups from a general population cross sectional study [25], which is commensurate with the current cohort's general age and state of health. The same was broadly true for CRP and fibrinogen [25].

**Table 1:** Baseline characteristics by incident primary combined nonfatal and fatal endpoint (*p*-value versus no event group).

Variable	Characteristic	Primary Endpoint (*n* = 861)	No Event (*n* = 4,792)	*p*-Value
**Continuous variables, mean (SD)**	Age (y)	75.8 (3.5)	75.3 (3.3)	<0.0001
	BMI (kg/m^2^)	26.9 (4.1)	26.8 (4.2)	0.52
	Systolic blood pressure (mmHg)	155.2 (22.8)	154.5 (21.6)	0.42
	Diastolic blood pressure (mmHg)	82.9 (11.8)	83.9 (11.3)	0.025
	Total cholesterol (mmol/l)	5.62 (0.87)	5.69 (0.91)	0.037
	HDL cholesterol (mmol/l)	1.24 (0.34)	1.29 (0.35)	<0.0001
	LDL cholesterol (mmol/l)	3.76 (0.76)	3.79 (0.80)	0.19
	Triglyceride (mmol/l)^a^	1.44 (1.53)	1.41 (1.51)	0.14
	IL-6 (pg/ml)^a^	2.91 (1.93)	2.61 (1.92)	<0.0001
	CRP (mg/l)^a^	3.64 (3.08)	3.01 (3.05)	<0.0001
	Fibrinogen (g/l)	3.62 (0.75)	3.59 (0.73)	0.31
**Categorical variables, ** ***n*** ** (%)**	Pravastatin	399 (46.3)	2,415 (50.4)	0.028
	Male	492 (57.1)	2,234 (46.6)	<0.0001
Smoking				0.088
	Never	267 (31.0)	1,649 (34.4)	
	Current smoker	229 (26.6)	1,283 (26.8)	
	Exsmoker	365 (42.4)	1,860 (38.8)	
Country				<0.0001
	Scotland	362 (42.0)	2,109 (44.0)	
	Ireland	382(44.4)	1,750 (36.5)	
	Netherlands	117 (13.6)	933 (19.5)	
History of:	Diabetes	129 (15.0)	474 (9.9)	<0.0001
	Hypertension	518 (60.2)	2,993 (62.2)	0.27
	Coronary disease	378 (43.9)	1,428 (29.8)	<0.0001
	Peripheral arterial disease	137 (15.9)	492 (10.3)	<0.0001
	Stroke or TIA	125 (14.5)	510 (10.6)	0.0009
	Any vascular disease	488 (56.7)	2,011 (42.0)	<0.0001

Please note that because of the design structure of the trial—recruiting more participants with hypertension/smokers and diabetes (and women) into the low risk primary prevention group—the significance or nonsignificance of univariate comparisons in this table could be potentially misleading. *p*-Values for continuous variables are from two-sample *t*-test and for categorical variables from chi-squared test.

a Values are geometric means (SD) calculated from the log-transformed distribution and the (*p*-value).

SD, standard deviation; TIA, transient ischemic attack.

### Associations of Elevated Inflammatory Markers with Primary Combined Nonfatal and Fatal Endpoints in the Cohort

When considering primary endpoints (including both fatal or nonfatal CVD events) comparing extreme thirds of the population in terms of plasma IL-6 levels after adjusting for only randomized treatment allocation gave an HR of 1.42 (95% CI 1.20–1.67). After adjusting for treatment allocation, age, gender, baseline LDL cholesterol, HDL cholesterol, triglycerides, systolic and diastolic blood pressure, current or exsmoker, BMI, diabetes, use of antihypertensive therapy, and country, the HR was attenuated to 1.20 (CI 1.01–1.42) comparing extreme thirds, and 1.10 (CI 1.03–1.18) for a 1-unit increase in log IL-6. As expected from results in Table 1, fibrinogen showed no significant association with risk of a primary endpoint (unpublished data). Associations of CRP with the primary endpoint have been previously reported [5].

### Dissecting Associations of Inflammatory Markers with Fatal and Nonfatal Events

We subsequently examined associations with risk for more specifically defined endpoints. Importantly, there was no difference in risk of any of the endpoints whether participants were randomized to pravastatin or placebo (see Table 2), allowing us to combine all study participants for the purposes of this analysis. Furthermore, there was no significant interaction with history of vascular disease at baseline (unpublished data). Nevertheless, existing CVD was adjusted for in full adjustment model.

**Table 2:** Associations of IL-6, CRP, and fibrinogen (HR for 1-unit increase in log IL-6, log CRP, and fibrinogen) of experiencing one of four categories of events by baseline treatment allocation.

Endpoints	IL-6 HR (95% CI)			CRP (HR 95% CI)			Fibrinogen (HR 95% CI)		
	Placebo (*n* = 2,839)	Pravastatin (*n* = 2,814)	*p*-Value	Placebo (*n* = 2,853)	Pravastatin (*n* = 2,827)	*p*-Value	Placebo (*n* = 2,819)	Pravastatin (*n* = 2,812)	*p*-Value
**Nonfatal CVD**	1.19 (1.02–1.39)	1.10 (0.97–1.34)	0.73	1.17 (1.07–1.29)	1.04 (0.94–1.14)	0.08	1.01 (0.88–1.17)	0.97 (0.84–1.13)	0.72
**Fatal CVD**	1.68 (1.30–2.18)	1.81 (1.34–2.45)	0.78	1.35 (1.14–1.60)	1.42 (1.17–1.74)	0.74	1.29 (1.02–1.64)	138 (1.05–1.81)	0.78
**Fatal other CV**	2.04 (1.14–3.67)	2.49 (1.37–4.53)	0.64	1.64 (1.12–2.42)	1.76 (1.17–2.63)	0.81	1.23 (0.71–2.14)	1.80 (1.06–3.05)	0.34
**Non-CVD mortality**	1.67 (1.33–2.10)	1.42 (1.14–1.77)	0.30	1.26 (1.08–1.46)	1.15 (1.00–1.32)	0.38	1.48 (1.22–1.79)	1.46 (1.21–1.77)	0.91

Event groupings as defined in the methods. Fatal CVD deaths preceded by nonfatal CVD are excluded. *p*-Value is for interaction between randomized treatment allocation and marker. Unadjusted HR (95% CIs) are presented.

**Figure 1:**
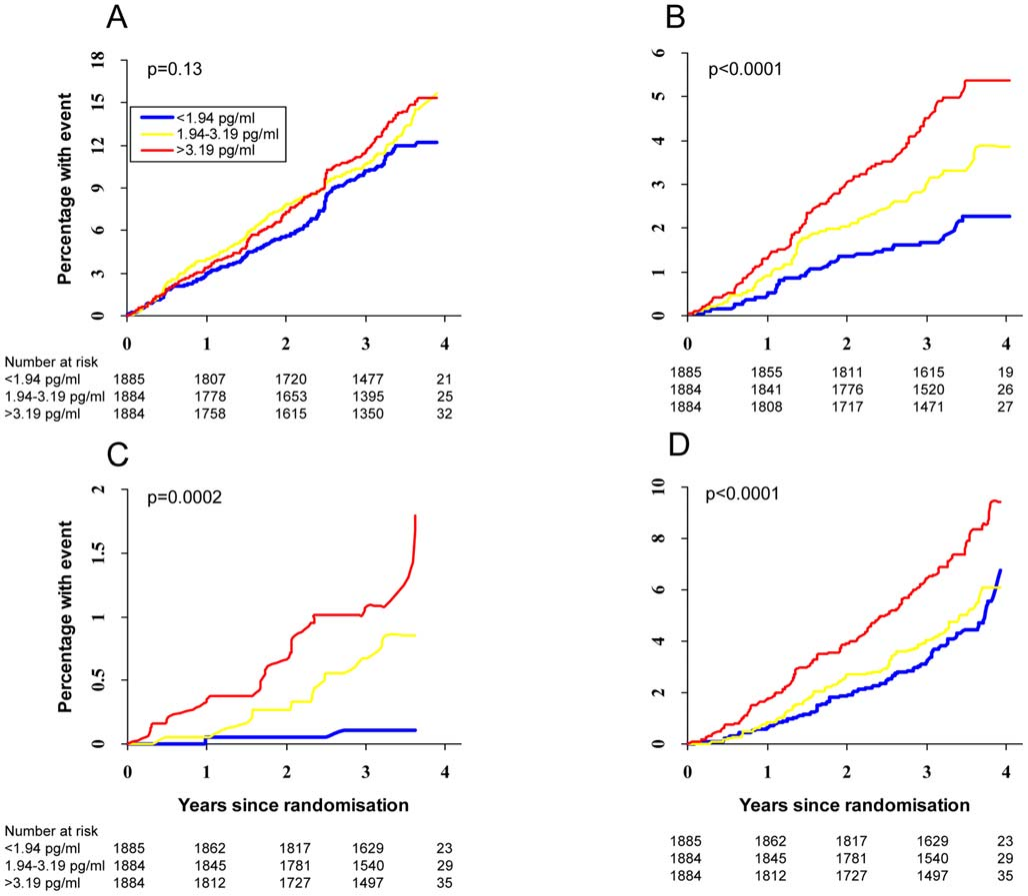
Kaplan-Meier time-to-event plots split by tertiles of IL-6 tertiles for (A) nonfatal CVD (*n* = 667 events), (B) fatal CVD (*n* = 189 deaths), (C) fatal other CV (*n* = 37 deaths), and (D) non-CVD mortality (*n* = 299 deaths).

Figure 1A–1D shows the Kaplan-Meier curves for cumulative percentage of events in the cohort by thirds of IL-6 expression for endpoints of nonfatal CVD, fatal CVD, fatal other CVD, and non-CV mortality. These curves clearly show that while thirds of IL-6 showed little discriminative ability in separating risk for nonfatal CVD in unadjusted models (*p* = 0.13), associations with risk for fatal CVD events, fatal other CV events, or non-CVD mortality were all highly significant (*p*≤0.0002 for all). Curves for CRP and fibrinogen showed broadly similar patterns (unpublished data), although IL-6 data showed clearest separation of curves.

**Figure 2:**
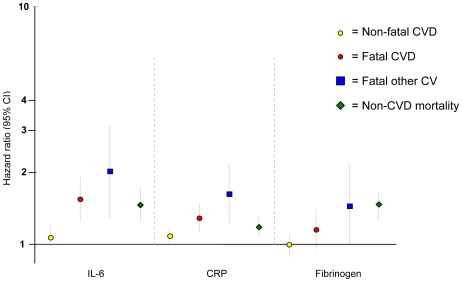
Plot showing associations of 1-unit increase in log IL-6, log CRP, and fibrinogen with HR of endpoints on a log scale (after adjusting for randomized treatment, age, gender, LDL cholesterol, HDL cholesterol, triglycerides, BMI, systolic and diastolic blood pressure, current and exsmoking, diabetes, previous CVD, use of antihypertensive therapy, and country).

Thereafter, HRs for associations of inflammatory markers with each of the four endpoint groups were assessed using continuous measures (to retain maximum power) without and with adjustment for all conventional risk factors plus other possible confounders (adjusted model detailed in Figure 2). As can be seen clearly, the HR for association of elevation in IL-6 with risk for nonfatal CVD of 1.06 (95% CI 0.94–1.20) was weaker than each of the mortality endpoints, as the upper confidence limits were less than the lower confidence limits of each of the mortality endpoints: HR 1.58 (1.28–1.94) for fatal CVD, 2.02 (1.28–3.18) for fatal other CV events, and 1.47 (1.24–1.74) for non-CVD death. Broadly similar associations were noted for CRP. For fibrinogen, there was no significant association with risk of nonfatal CVD, but borderline significant associations were evident with each of the CVD mortality endpoints (Table 3). Competing risk models combining nonfatal CVD and fatal CVD events were then constructed to formally test the differences in HRs for IL-6, CRP, and fibrinogen. For models adjusting for randomized treatment the HRs for fatal CVD were found to be significantly higher (χ^2^ for heterogeneity = 14.1, *p* = 0.0009; χ^2^ = 12.0, *p* = 0.0025; χ^2^ = 8.7, *p* = 0.013 for IL-6, CRP, and fibrinogen, respectively). Significant differences were also observed for fully adjusted models. Finally, we checked whether white cell count also showed the same pattern as the other three inflammatory markers and once again noted much stronger associations with fatal CVD events (data on request).

**Table 3:** Associations of IL-6, CRP, and fibrinogen with risk (HR for 1-unit increase in log IL-6, log CRP, or fibrinogen) of experiencing one of the four categories of events.

Inflammatory Marker	Model	Nonfatal CVD (*n*≥667 Events)		Fatal CVD (*n*≥189 Deaths)		Fatal Other CV (*n*≥37 Deaths)		Non-CVD Mortality (*n*≥299 Deaths)	
		HR	95% CI	HR	95% CI	HR	95% CI	HR	95% CI
**IL-6 (** ***n*** ** = 5,653)**	**A**	1.17	1.04–1.31	1.75	1.44–2.12	2.25	1.48–3.41	1.54	1.31–1.80
	**B**	1.07	0.95–1.21	1.57	1.27–1.94	2.13	1.38–3.31	1.46	1.24–1.72
	**C**	1.06	0.94–1.20	1.58	1.28–1.94	2.02	1.28–3.18	1.47	1.24–1.74
**CRP (** ***n*** ** = 5,680)**	**A**	1.11	1.03–1.18	1.39	1.22–1.58	1.70	1.28–1.69	1.20	1.08–1.33
	**B**	1.08	1.01–1.16	1.30	1.14–1.49	1.70	1.28–2.25	1.18	1.07–1.31
	**C**	1.08	1.00–1.16	1.32	1.16–1.51	1.63	1.21–2.18	1.19	1.08–1.32
**Fibrinogen (** ***n*** ** = 5,631)**	**A**	0.99	0.90–1.10	1.33	1.11–1.58	1.48	1.01–1.61	1.47	1.29–1.68
	**B**	1.01	0.91–1.13	1.20	1.00–1.45	1.45	0.98–2.15	1.46	1.27–1.68
	**C**	1.01	0.91–1.12	1.19	0.99–1.44	1.45	0.97–2.17	1.47	1.27–1.68

Event groupings as defined in the methods. Fatal CVD deaths preceded by nonfatal CVD are excluded. Model A, adjusted for randomized treatment. Model B, adjusted for randomized treatment, age, gender, LDL cholesterol, HDL cholesterol, systolic blood pressure, current smoker, diabetes, previous CVD (CHD, stroke, peripheral arterial disease, stroke, and transient ischaemic attack), use of antihypertensive therapy, and country. Model C, Model B+ adjusted for log triglyceride, BMI, diastolic blood pressure, exsmoker (as well as current smoker).

### Prediction of Fatal and Nonfatal CVD Events

We explored what the differing associations of inflammatory markers with risk of fatal versus nonfatal CVD events might mean for risk prediction by constructing C-statistics relating to the predictive ability of traditional risk factors with and without IL-6 and CRP for the clinically relevant endpoints of nonfatal CVD, fatal CVD, and fatal other CVD. Conventional risk factors predicted risk of fatal CVD (C-statistic 0.699) and fatal other CVD (0.698) to a similar degree, although the predictive ability for nonfatal CVD was lower (0.630). Addition of IL-6 to traditional risk factors did not significantly increase discriminative predictive ability in the nonfatal CVD group (*p* = 0.20), although CRP did so modestly (+0.003, *p* = 0.023). However both IL-6 and CRP added greater discriminative ability to the fatal CVD (+0.017 and +0.015; both *p*<0.0001) and fatal other CVD groups (+0.047 and +0.075; both *p*<0.0001) (full data on request).

## Discussion

To our knowledge, this is the first study to directly and simultaneously compare prospectively the associations of a range of inflammatory markers separately with risks of fatal and nonfatal CVD events in a hypothesis driven manner. The results show that IL-6, CRP, and to a lesser extent, fibrinogen are more closely linked to risk of fatal MI or stroke (i.e., fatal CVD) than to nonfatal vascular events in the elderly at risk. In other words, we suggest that inflammatory markers are linked more closely to risk of death from CVD causes than to risk of nonfatal CVD events. We believe our findings may be a key feature of the inflammatory-vascular risk paradigm, which has been previously overlooked in the literature, but one that may have both important biological, clinical, and health implications.

### PROSPER Results in Context of the Literature

Our key observation of a stronger link of inflammatory markers with fatal CVD compared to nonfatal CVD significantly extends suggestive (but inconclusive) observations from previous reports, including studies in middle-aged and elderly individuals [4],[11]–[20],[26],[27], of potentially stronger associations between inflammatory markers with CVD death. While the body of evidence from these other studies has alluded to this possibility, our study is the first we are aware of to systematically and simultaneously show inflammatory markers (including IL-6 and CRP) are more strongly associated with fatal than nonfatal vascular events. We therefore suggest that combining both fatal and nonfatal CVD events as a single endpoint may make inappropriate assumptions about a common aetiology, whereas in reality subtle but important differences in risk factor patterns, perhaps particularly with regard to inflammatory markers, exist.

We note that the significantly stronger association with fatal CVD was particularly marked for IL-6 in PROSPER, which is a novel observation. Our results for fibrinogen are consistent with and significantly extend a previous report from the Fibrinogen Studies Collaboration; the results section reports a borderline (*p* = 0.05) higher HR of 2.68 (95% CI 2.36–3.03) per 1 g/l increase in fibrinogen for risk for fatal CHD compared with an HR of 2.30 (95% CI 2.10–2.52) for risk of a nonfatal MI [4], but there was no further discussion on this finding. Interestingly, we found fibrinogen to be the least discriminatory between fatal and nonfatal CVD, and IL-6 to be most discriminatory. A similar collaborative meta-analysis of individual prospective data for CRP did not explore such differences, but does discuss the possibility of early publication bias in the CRP literature [2]. Of note, earlier prospective studies for CRP were more likely to include only fatal CVD endpoints than later studies [15],[16],[19], perhaps helping to explain some of the stronger associations seen for CRP with CVD outcomes in earlier reports. Furthermore, this phenomenon may not be limited to traditional inflammatory markers of the acute phase response. For instance, neopterin, a marker of macrophage activation, also showed trends to be more strongly related to risk of death alone (HR for upper quarter versus lower three quarters, 1.86 (95% CI 1.24–2.77) versus its association with acute MI (HR 1.35, CI 1.03–1.79) in a population with prevalent vascular disease [28].

### Aetiological Relevance

One interpretation of our findings is a possible mechanistic role for inflammation in the promotion of serious vascular disease, which is more likely to lead to death. Inflammation marker levels are associated with risk of death in the acute setting [29],[30], and it may be that elevated baseline inflammation increases the risk (or is associated with an increase in risk) that leads to more incident vascular events being fatal. The mechanisms for this observation require further study, but could include chronic low-grade inflammation being associated with a lowered buffering capacity to cope with the stress of a significant vascular insult, a greater likelihood of any vascular insult to be accompanied by a fatal cardiac arrhythmia or more diffuse myocardial necrosis [31], or indeed a greater clotting potential, or a combination of mechanisms. The search for such mechanisms will be stimulated by our findings. Among the inflammatory markers IL-6 seemed to show more marked associations with fatal CVD than CRP or fibrinogen, although epidemiological studies alone cannot infer causality. The recent availability of IL-6 blocking agents in autoimmune diseases [32] may help promote relevant research in this area and there is increasing need to test whether inflammation blockers lessen vascular risk, or otherwise, in a range of differing populations. Alternatively, inflammatory markers may not be causally related to vascular death; our findings could also be consistent with the hypothesis that elevated levels of inflammatory markers are an indicator of subclinical disease (vascular or otherwise) and lifestyle circumstances that promote death from several causes [26]. To this end all inflammatory markers were, as expected, also independently associated with non-CVD mortality.

### Clinical Relevance

CRP as a CVD risk factor is once again in sharp focus post JUPITER trial [33],[34], but there is continued debate about its inclusion in CVD risk assessment [35]. However, the differential pattern of association of CRP (and IL-6) with fatal versus nonfatal events is not at all considered as a potentially important feature in prior literature, but may need consideration by relevant guideline committees. For example, recently devised SCORE risk charts for European-wide use, devised by a European guidelines committee [36], is designed to predict risk of 10-y CVD fatality rather than combined fatal and nonfatal CVD. Thus, the use of CRP or other inflammatory markers in CVD risk assessment, if ever widely adopted, may well be more suited to prediction of fatal CVD events. For instance, Zethelius et al. recently demonstrated significant improved ability of four biomarkers including CRP (others NT-proBNP, cystatin C, and troponin I) to usefully enhance prediction of CVD death beyond traditional risk factors [37]. However, these authors appeared not to be aware of the possibility that their superior predictive results may have been influenced by their use of only a fatal CVD endpoint. Accordingly, our findings may help improve future research of novel CVD risk factors for clinical use. Of course the exploration of our conclusions, if confirmed, should help also in maintaining future health.

Our findings may also hold particular relevance to patients with rheumatoid arthritis (RA), a chronic inflammatory condition, recently shown to be linked to a greater likelihood of sudden death compared to non-RA patients [38], and a greater 30-d case fatality rate following MI [39]. Thus, improved understanding of the mechanisms linking inflammation markers to fatal CVD events may ultimately improve clinical management not only in the low grade setting but also in the high grade inflammation arena of auto-immune conditions. In this respect, IL-6 blocking agents are now licensed for clinical use in RA [32], and are likely to be used in future large trials to tease out causal pathways and net vascular effects.

### Generalisability of PROSPER

We examined the dataset for peculiarities that might render our findings atypical, but found no such evidence. First, the baseline associations of inflammatory markers with each other and with other risk factors in this population are broadly in keeping with expectations. Second, the observed adjusted risk associations of IL-6 and CRP with the combined nonfatal and fatal CVD endpoint are broadly consistent with findings from recent important meta-analyses including younger populations [2],[3]. Similarly, PROSPER findings on other risk factors (e.g., leptin [40]) or risk criteria (e.g., metabolic syndrome [41]) are also in line with observational studies conducted in younger populations [41],[42]. Third, our findings for CRP in terms of modest improvement in prediction of the traditionally examined combined fatal and nonfatal CVD endpoint, beyond conventional risk factors, are in line with recent reports from the Cardiovascular Health study group [10] and the MONICA Augsburg study [9] where this variable only modestly improved prediction of CVD events beyond that of conventional risk factors. Fourth, all three inflammatory markers, measured independently, showed similar associations. Fifth, our findings for IL-6 and CRP were similar in unadjusted and adjusted analysis and thus unlikely to be biased by adjustment by other CVD risk factors. Sixth, the results were also not influenced by randomized treatment. Finally, the endpoint ascertainments were of high quality given that PROSPER was a clinical trial with adjudication committees with rigorous endpoint criteria.

### Study Limitations

Although this is a large study with a large number of events, it has insufficient power to further analyse the “fatal other CVD” and “non-CVD mortality” groups by subgroups of events such as MI versus stroke. The study comprised elderly at risk patients (largely secondary prevention), and we cannot exclude the possibility that this group may show more marked differences in the magnitude of association of inflammation markers with fatal versus nonfatal CVD events as compared with studies in middle-aged populations, although, as discussed above, the extent of other data suggests PROSPER findings are generally consistent with those in younger cohorts. Although PROSPER is a high vascular risk population, participants were otherwise clinically healthy, as per trial exclusion criteria (e.g., no diagnosed prevalent cancers at baseline [21],[22]). The study only had up to an average of 3.2-y follow-up, although short follow-up may be appropriate in elderly populations. We recognize the study is more relevant to reveal novel associations rather than test prediction but nevertheless, on the basis of our findings others should now retest their risk factor data to check for differential prediction of fatal versus nonfatal events. The study was conducted in the context of a randomized trial of statins [22]—however, there was no interaction between active and placebo group status for the main analyses in the present report. We did not adjust for regression dilution because of lack of repeat measurement for parameters but note that IL-6 associations with fatal CVD events in particular may be considerably strengthened since this molecule exhibits greater intra-individual variability in its circulating concentrations than does CRP. In this regard, that baseline IL-6 levels demonstrated the greatest differential association between fatal and nonfatal CVD events is remarkable, and of potentially considerable clinical significance. IL-6 is also a more credible causal factor for vascular disease than is CRP. Finally, we recognise that although our findings were consistent for all three inflammatory markers, others will need to replicate our work in prospective cohorts that have all the required endpoints and baseline measurements. In addition, further validation by meta-analysis [43] may soon be possible, whereas Mendelian randomization techniques could be used to examine for potential causal associations [44],[45].

### Conclusion

We show that three markers of low grade inflammation (in particular IL-6 and CRP), are more strongly associated with fatal CVD events than nonfatal vascular events. This novel observation has potentially important aetiological and clinical implications. Future studies should, wherever possible, systematically compare and contrast risk associations of biomarkers (new and old) with fatal versus nonfatal CVD events.

## Abbreviations

BMI: body mass index
CHD: coronary heart disease
CI: confidence interval
CRP: C-reactive protein
CVD: cardiovascular disease
HDL: high-density lipoprotein
HR: hazard ratio
IL-6: interleukin-6
LDL: low-density lipoprotein
MI: myocardial infarction
